# An Adaptive Evolutionary Algorithm for Traveling Salesman Problem with Precedence Constraints

**DOI:** 10.1155/2014/313767

**Published:** 2014-02-17

**Authors:** Jinmo Sung, Bongju Jeong

**Affiliations:** Department of Information & Industrial Engineering, Yonsei University, 50 Yonsei-Ro, Seodaemaun-gu, Seoul 120-749, Republic of Korea

## Abstract

Traveling sales man problem with precedence constraints is one of the most notorious problems in terms of the efficiency of its solution approach, even though it has very wide range of industrial applications. We propose a new evolutionary algorithm to efficiently obtain good solutions by improving the search process. Our genetic operators guarantee the feasibility of solutions over the generations of population, which significantly improves the computational efficiency even when it is combined with our flexible adaptive searching strategy. The efficiency of the algorithm is investigated by computational experiments.

## 1. Introduction

In most business processes, a set of operations or activities are constrained by precedence relationships that are imposed by technological characteristics of products or services. The precedence constraints represent a typical operational structure constrained by sequentially and/or related operations. Therefore, the determination of operation sequences considering precedence constraints is one of the most important issues in many industrial problems such as production planning, scheduling, and project network planning problems. In most practices, however, sequencing problem is very difficult to solve because of its combinatorial complexity. More often than not, the solution may be impractical to be used in real environment due to system variability, while most managers in charge of operation sequencing want more sturdy and robust solutions. This study considers, specifically, traveling salesman problem with precedent constraints (TSPPC) and aims to obtain more robust and efficient operation sequence with minimum setups.

TSPPC is a variant of traveling salesman problem (TSP) because all nodes should be visited once but in predetermined order. Precedence constraints make TSPPC have the wider range of industrial applications such as scheduling, project management, and process routing. In solution methods, however, TSP is known to be a class of NP-hard problem and TSPPC is even more complicated with additional constraints. Since no exact solution can be obtained in reasonable computational time and a good solution needs to be practical and implementable in real environment, careful development of new solution method is crucial. As a solution technique, we will consider the evolutionary algorithm which is proven to be very effective in large scale of solution space and provides high flexibility in searching strategy.

We will first present a mathematical model of TSPCP as an appropriate network flow model. A new evolutionary algorithm will be introduced and in subsequent sections modifications of algorithm will be proposed for further improvement of solution. Finally, experimental analyses will validate the efficiency of our algorithms.

## 2. Literature Review

Abundant researchers have been interested in various types of sequencing problem using TSP. Reinelt [1] developed a traveling salesman problem library (TSPLIB) which is meant to provide researchers with a broad set of test problems from various sources and with various properties. Chen [2] discussed an AND/OR precedence constrained sequencing problem and formulated it as a state-constrained traveling salesman model and applied the model to assembly scheduling problem. Also, He and Kusiak [3] developed a mixed integer formulation and a simple and easy heuristic for a single machine scheduling problem with sequence-dependent setup times and precedence constraints. Recently, Su [4] proposed a unique reasoning method supported by an artificial intelligent technique of case-based reasoning with evolutionary algorithm to solve the TSPPC problem. Lee et al. [5] suggested a tree-structured precedence graph to solve the problem of selecting and sequencing operations in process planning with the objectives of minimizing the sum of operation processing, setup, and tool change costs with precedence constraints. Lambert [6, 7] formulated a disassembly scheduling problem by modification of two-commodity network flow model including TSPPC problem. Mingozzi et al. [8] proposed an exact algorithm to solve the problem using dynamic programming and bounding functions to reduce state space graph. Ascheuer et al. [9] described the implementation of a branch and cut-algorithm and gave computational results for real-world instances and benchmark problems from TSPLIB. Many exact and heuristic algorithms have been developed in the field of operations research (OR) to solve the variants of TSP. However, precedent constraints always bother the researchers in making efficient algorithms because with them the problems become dramatically difficult.

Many researched also used the memetic computational algorithms to solve TSP. Jati and Suyanto [10] used the firefly algorithm with the discrete distance between two fireflies and the movement scheme. Ouaarab et al. [11] employed cuckoo search (CS) algorithm inspired by the breeding behavior of cuckoos. They extended and improved CS by reconstructing its population and introducing a new category of cuckoos. In spite of using memetic computation, precedent constraints are still challenging in TSP.

Meanwhile, genetic algorithm is widely accepted as efficient algorithm to be applied in many intractable problems like TSP. Zeng et al. [12] used genetic algorithm for antenna design. Poland et al. [13] solved exosensor distribution optimization problem using genetic algorithm to generate globally optimal sensor distributions for a smart home replica kitchen.

Some researchers used genetic algorithms to tackle TSPPC. As one of the first researchers using genetic algorithm for this problem, Potvin [14] introduced genetic algorithms for traveling salesman problem and its extensions. Recently, more efficient genetic algorithms were developed using some additional techniques such as special decoder, penalty function, and special genetic operator, all of which efficiently generate good feasible solutions by Michalewicz and Fogel [15]. Ghazalli and Murata [16] used genetic algorithm to find optimal disassembly sequences for disassembling the end-of-life product. He used topological sort method to generate feasible solutions and fix infeasible solutions. Moon et al. [17–19] also proposed a topological sort based evolutionary approach to solve TSPPC and tool selection problem. Yun and Moon [20] also used a similar technique to solve precedence-constrained sequencing problem. Even though most of these approaches are very simple and easy to implement, a major drawback is that the quality of solution is not satisfactory enough because of randomness of solution generation. Avoiding randomness and guiding to the better choice of solution are essential to improve GA based algorithms.

In this study, we propose an adaptive evolutionary algorithm to improve the computational efficiency and the quality of solutions. The proposed algorithm searches only the feasible solution space by adopting efficient genetic operators and employs adaptive search strategies to adaptively apply the genetic operations on the current population. Throughout this paper, we use “evolutionary algorithm” as the same meaning as “genetic algorithm,” “evolutionary strategies,” and “evolutionary programming,” which are found in many literatures. The objective of this study is to develop an efficient evolutionary process scheme for TSPPC. The algorithm seeks a solution to minimize the total processing time for implementing all the operations with sequence dependent setup times.

## 3. Mathematical Model of Traveling Salesman Problem with Precedent Constraints (TSPPC)

The mathematical model of TSPPC is quite similar to the traveling salesman problem (TSP). The *G* = (*V*, *A*) graph is used to define TSPPC, where *V* = {0, 1,…, *n*} and *A* = {(*i*, *j*) | *i*, *j* ∈ *V*} which indicate nodes and arcs, respectively, in graph *G*. In TSPPC, a node corresponds to an operation and an arc to a processing time. To formulate TSPPC, we need to modify the two-commodity network flow model (as discussed by Lambert [6]) used in TSP as follows.

*TSP*. The model assumes that each node has one demand unit and the starting node has *N* units to meet the demand of each node. In other words, the salesman starts with *N* units at the starting node and travels to the end node till meeting the demand of all nodes. The simplified commodity network flow model considers only the amounts of belonging when the salesman enters or leaves a node. Then, a mathematical model can be formulated as follows:

(i) parameters
  *N*: the number of nodes;
  *t*_*ij*_: the distance from node *i* to node *j*;
(ii) decision variables
  *a*_*k*_: integer variable which is decreasing aggregate counter;
    (1) $$\begin{array}{c} x_{ij}:\{\begin{array}{cc} 1, & \text{if node }j\text{ is visited next to}\;i\\ 0, & \text{otherwise;} \end{array} \end{array}$$
  *p*_*ij*_: decreasing partial counter which is decreased with 1 if node *j* is visited next to *i*;
(iii) objective
  (2) $$\begin{array}{c} \text{Minimize}\sum_{ij}t_{ij}\times x_{ij}; \end{array}$$
(iv) subject to
  (3) $$\begin{array}{c} \sum_{j}x_{ij}=1\;\forall i, \end{array}$$
  (4) $$\begin{array}{c} \sum_{i}x_{ij}=1\;\forall j, \end{array}$$
  (5) $$\begin{array}{c} \sum_{i}p_{ij}=a_{j}\;\forall j, \end{array}$$
  (6) $$\begin{array}{c} \sum_{i}p_{i0}=N,\;\;\sum_{i}p_{0i}=0,\;\;p_{jj}=0\;\forall j, \end{array}$$
  (7) $$\begin{array}{c} p_{ij}\leq \left(N+1\right)\times x_{ij}\;\forall i,j, \end{array}$$
  (8) $$\begin{array}{c} \sum_{j}p_{ij}=a_{i}-1\;\forall i, \end{array}$$
  (9) $$\begin{array}{c} x_{ij}=\text{binary},\;a_{i}=\text{integer}\;\forall i,j. \end{array}$$

The objective function (2) is to minimize total traveling distance in TSP, for example, the total processing time of operations in scheduling problem. Constraints (3) and (4) indicate that all nodes have one preceding and one subsequent node. Constraint (5) represents counter aggregation, where the amount of belonging at each node is calculated by sum of preceding belongings. The total belonging size is restricted by constraint (6). And constraint (6) also makes starting and end condition for the start and end point. Constraint (7) couples counter and flows. Constraint (8) is for counter decrement. The last constraint (9) restricts the decision variables to integer.

Then TSPPC is formulated as follows. 

*TSPPC*. TSPPC has the same constraint sets of TSP and one additional constraint set for precedence relationships between node *i* and *j* as follows:

(10) $$\begin{array}{c} a_{i}>a_{j}. \end{array}$$

Constraint (10) indicates that node *i* precedes node *j*.

## 4. Algorithm

**Figure 1:**
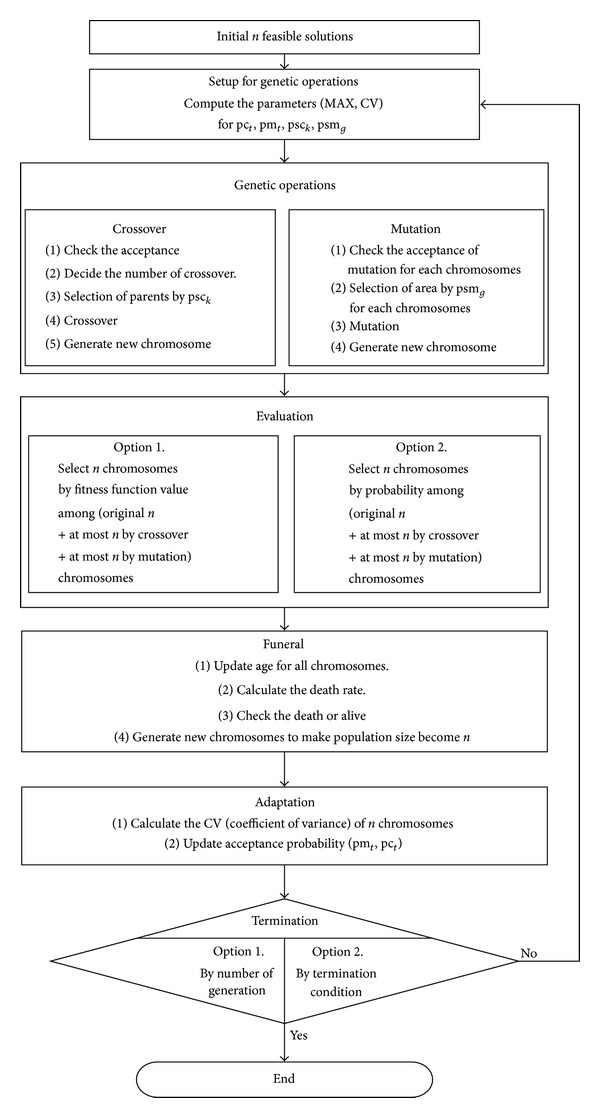
Procedure of the adaptive evolutionary algorithm.

This section presents our proposed evolutionary method which searches only feasible solution spaces to improve the computational efficiency. The overall procedure of the algorithm is shown in Figure 1. The procedure includes crossover and mutation processes along with adaptive search strategies. The concept of the adaptive scheme is to adaptively change the values of parameters to enhance the search efficiency.

The details of algorithms are described in the following subsections.

### 4.1. Solution Representation

**Figure 2:**
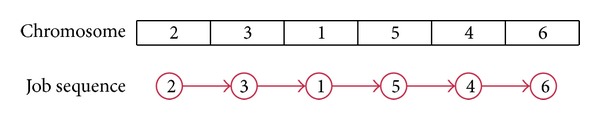
Representation of chromosome.

A chromosome representing the sequence of operations is shown in Figure 2. Data are represented using the linked-list format for the evolutionary algorithm (Horowitz et al. [21], Michalewicz [22]).

Since each gene corresponds to an operation, Figure 2 represents an operation sequence as 2-3-1-5-4-6. Also we have information of the precedence constraints as *n* × *n* matrix with parameters pre_*ij*_. If pre_*ij*_ = 1, then *i* operation should be done before *j* operation.

### 4.2. Initial Solution and Parameter Setup

The *n* initial solutions can be generated by topological sort (Moon et al. [19], Yun and Moon [20]). All solutions of the population are evaluated by the degree of fitness. In terms of TSP, the objective is to find a visit sequence with the shortest traveling time through all nodes. The fitness value of each solution is computed as follows:

(11) $$\begin{array}{c} \text{fi}{\text{t}}_{k}=\sum_{ij}t_{ij}\times x_{ijk}, \end{array}$$

where fit_*k*_ is the fitness-function value of *k*th chromosome, *t*_*ij*_ indicates the setup time of changing operation *i* to operation *j*, and *x*_*ijk*_ is a binary variable for *k*th chromosome with a value of 1 if node *i* precedes node *j* and 0 otherwise.

We have four parameters to be set for our algorithm: crossover acceptance probability (pc_0_), crossover selection probability (psc_*k*_), mutation acceptance probability (pm_0_), and mutation selection probability (psm_*gk*_).

The crossover acceptance probability is used to obtain the number of crossover operations over the current population. Similarly, the mutation acceptance probability is used to decide whether a chromosome accepts mutation operation or not and consequently is used to obtain the number of mutation operations over the current population. The crossover selection probability is a probability of selecting a chromosome *k* (as a parent chromosome) on which crossover operation is performed and computed as follows:

(12) $$\begin{array}{c} \text{ps}{\text{c}}_{k}=\frac{\left(\mathrm{Max}-\text{fi}{\text{t}}_{k}\right)}{\sum_{c}\left(\mathrm{Max}-\text{fi}{\text{t}}_{c}\right)}, \end{array}$$

where Max is the maximum fit_*k*_ (fitness-function value) among *n* chromosomes.

According to (12), a chromosome with the low fitness-function value has a high probability for selection.

The mutation selection probability is a probability of selecting a gene (as both end points) in chromosome *k* to determine the mutation region and computed is follows:

(13) $$\begin{array}{c} \text{ps}{\text{m}}_{gk}=\frac{t_{\left[g-1][g\right]}+t_{\left[g][g+1\right]}}{\text{fi}{\text{t}}_{k}}, \end{array}$$

where psm_*gk*_ is the probability to select *g*th gene as a start or end point of mutation region. [*i*] means the value of *i*th gene. For example, *t*[1][2] with operation sequence 2-5-3-4-6-1 indicates the setup time of changing operation 2 to operation 5. If a gene is the first or the last one, calculate one side value.

These all parameter values are computed before the algorithm proceeds.

### 4.3. Genetic Operations

Genetic operations are designed to search only feasible solutions over the entire generations. The modified topological sort that merges two instances into one is considered to generate feasible chromosomes. We can use pc_0_ and pm_0_, which are the probabilities of crossover and mutation, respectively.

#### 4.3.1. Crossover

A crossover is operated according to the value of pc_0_, which is a crossover probability. If the crossover is accepted, two chromosomes will be selected for crossover. With a cumulative probability of crossover selection probabilities of each chromosome, that is, cumulative psc_*k*_, two chromosomes are chosen using a random number generator. Thus, the higher chance of selection goes to a chromosome with the higher crossover selection probability. After two chromosomes are selected in this manner, an offspring through crossover operation on the chromosome is created by incremental inclusion of “selectable” nodes. A node is selectable if all precedent nodes are already included in the current chromosome or no precedent node exists. Our procedure maintains a selectable node set, *E*, until the set is empty. Let *L* be the length of a chromosome and let *l* be the position of genes in the chromosome. The crossover procedure is described as follows.

**Step 1**

Create a graph *G* = (*N*, *A*) of TSPPC. Set *l* = 1

**Step 2**

Create a selectable node set *E* from *G*.

**Step 3**

Select two *l*th genes from both parent chromosomes. We have four possible cases as follows:

Case 1. Two selected genes are different and found in *E*. Select one arbitrarily.
Case 2. Two selected genes are same and found in *E*. Then select that one.
Case 3. Only one gene is selected and found in *E*. Then select that one.
 (This happens when a gene has been removed in the previous iteration.
Case 4. No selected genes are found in *E*. Then select a gene (node) from *E* arbitrarily.

**Step 4 (update *G*)**

Delete the selected node in Step 3 with the corresponding arcs.

**Step 5**

If *l* = *L*, terminate. Otherwise, set *l* = *l* + 1 and go to Step 2.

Since all genes in a new chromosome are selected from the selectable node set *E*, the precedent constraints are always satisfied, which means the crossover operations search are always the feasible solution spaces. Over the crossover operations, the maximum *n* chromosomes are newly generated from *n* original chromosomes,

#### 4.3.2. Mutation

A mutation is performed on a chromosome in order to create, by chance, an unexpected good solution. In our algorithm, the mutation is operated according to the value of pm_0_, mutation acceptance probability. If mutation is accepted, a mutation region is determined so that the mutation is performed on an interval between two genes. With a cumulative probability of mutation selection probabilities of each gene of given chromosome, that is, cumulative psm_*gk*_, two genes are chosen using a random number generator. This means that the higher chance of selection goes to a gene with the higher mutation selection probability.

After selecting a pair of genes, all genes between the pair are topologically sorted and subject to only their precedence constraints. It is obvious that this mutation operation keeps the feasibility of newly created chromosome because the front part (or the latter part) of the mutation region satisfies the precedence constraints with the mutation region in the original chromosome, and even the mutation region is replaced with other feasible region, the feasibility is still maintained.

The mutation operations create newly maximum *n* chromosomes with *n* original chromosomes,

### 4.4. Termination Criterion

We have two options to terminate the algorithm. The first one is to set the fixed number of generations, which is easy and popular way. The other criterion is to use the best fitness function value of each generation and check the trend of improvement over the generations. If the best fitness function value at generation *t*(min⁡fit_*t*_) is not changed for the certain number of generations, the algorithm stops. This is represented as follows:

(14) $$\begin{array}{c} \min{\text{fit}}_{t}=\min{\text{fit}}_{t+1}=\cdots =\min{\text{fit}}_{t+R}, \end{array}$$

where min⁡fit_*t*_ is the best fitness-function value at the *t*th generation.

If termination criterion is not satisfied, the algorithm selects *n* chromosomes for the next generation. We may have two options for selection. Firstly, we can choose *n* chromosomes by evaluating the fitness function values among all chromosomes from the original, crossover, and mutation operations. The second way for selection is to use the probability of each chromosome. The chromosome with lower fit_*t*_-value has the higher probability for selection as follows:

(15) $$\begin{array}{c} \text{psx}{\text{g}}_{k}=\frac{\mathrm{Max}-\text{fi}{\text{t}}_{k}}{\sum_{i}\left(\mathrm{Max}-\text{fi}{\text{t}}_{i}\right)}, \end{array}$$

where psxg_*k*_ is the probability that the *k*th chromosome is selected for next generation.

### 4.5. The Aging of Chromosome

As generations continue, some survival chromosomes are getting older. All organisms experience aging process and may have a peak time to achieve the best performance. We can employ this natural property to improve the searching spaces. With a lifespan value, funeral probability is computed as follows:

(16) $$\begin{array}{c} \text{fn}{\text{r}}_{k}=1-e^{-\text{ag}{\text{e}}_{k}/\text{als}}, \end{array}$$

where fnr_*k*_ is the probability of death and age_*k*_ is the age of *k*th chromosome and als is the average lifespan.

A chromosome with the higher funeral probability has the lower chance of survival over the next generation.

### 4.6. Adaptive Search Strategy

In evolutionary algorithm, the population of generation changes as the generations proceeds. In order to obtain the better solutions, the better population needs to be generated at each generation. We can use this idea to create the search strategy for getting the better solution spaces. Due to the necessity of adaptability to each generation, we use a term of “adaptive” strategy.

For development of adaptive search strategy, we need to identify the characteristics of population. The parameters used in our algorithm can be quite useful to do so. Basically, new chromosomes are created by genetic operations, that is, crossover and mutation operations. This means that modifying the parameters of genetic operations makes the algorithm have the adaptability. We consider the crossover and mutation acceptance probabilities as the parameters for the algorithm to have the adaptability.  Table 1 shows how we change the two parameters according to the number of generations and variance of fitness function values of the current population.

Without loss of generality, in evolutionary algorithm we assume that as the number of generations increases, the chance of finding the better solution also increases. In this context, we think that as the number of generations increases, the more crossover operations are necessary because the better solution can be obtained from the more crossover among them, while the number of mutation operations need to be reduced in order to avoid unnecessary solution regions. At the same time, we look into the distribution of fitness function values of chromosomes. If the variance of the fitness function values is high, we need more crossover operations on the population to get the unexplored solutions in the current solution region. In this case, however, the mutation operation does not have to be encouraged in order to avoid unnecessary solution search. We have the opposite statements in case of high variance of fitness function value in population.

The above adaptive search strategies can be simply implemented with the following parameters:

(17) $$\begin{array}{c} \text{p}{\text{c}}_{t}={\left(\text{p}{\text{c}}_{0}\right)}^{\alpha \times t\times \text{C}{\text{V}}_{t}}, \end{array}$$

(18) $$\begin{array}{c} \text{p}{\text{m}}_{t}={\left(\text{p}{\text{m}}_{0}\right)}^{\beta /t\times \text{C}{\text{V}}_{t}}, \end{array}$$

where pc_*t*_ and pm_*t*_ are the acceptance probabilities of crossover and mutation at *t*th generation, respectively. *α* and *β* are the parameters reflecting the characteristics of problem. CV_*t*_ is coefficient of variance of population at *t*th generation.

## 5. Computational Experiments

In this section, we present the results of our experimentation with the proposed algorithm. We investigate the efficiency of genetic operations that avoid the generation of infeasible solution in the next subsection, the behavior of adaptive strategies, and then compare the results against other algorithm, and finally, where possible, the results are compared with the best known solutions.

### 5.1. Efficiency of Feasible Solution Search

**Figure 3:**
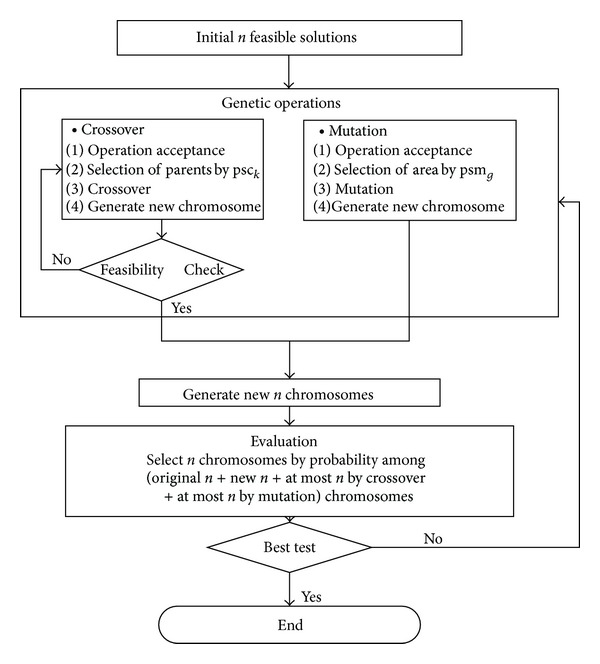
The procedure of a general approach that uses the separate feasibility check module.

Our approach uses the topological sort and special genetic operation procedures to generate feasible populations. In order to focus on our genetic operations that guarantee the feasibility of solution, we present a general approach that uses a separate feasibility check module and compare the computation results. The overall procedure of the general approach is shown in Figure 3.

For experiments, the initial parameters are given as pm_0_ = 0.8, *α* = 1, and *β* = 1. The experiments are performed on micro-PC with 3.0 GHz processor and 2 GB RAM.  Table 2 shows the results of the general approach and our adaptive evolutionary algorithm.

From the results, we observe that two approaches find the best solutions for the small size examples. However, our algorithm is far efficient in computation time for especially large size problems, which is because our algorithm explores only the limited feasible solution space. Also our algorithm shows much better solution results than the general approach. The computation time of the general approach significantly increases as the size of problem increases. This is because the crossover operations on the infeasible chromosomes generate infeasible chromosomes again and it repeats without improving the solution. Since the probability of infeasibility increases as the number of nodes increases, the general approach is getting worse for the large size problems.

### 5.2. Behavior of Adaptive Search Strategy

**Figure 4:**
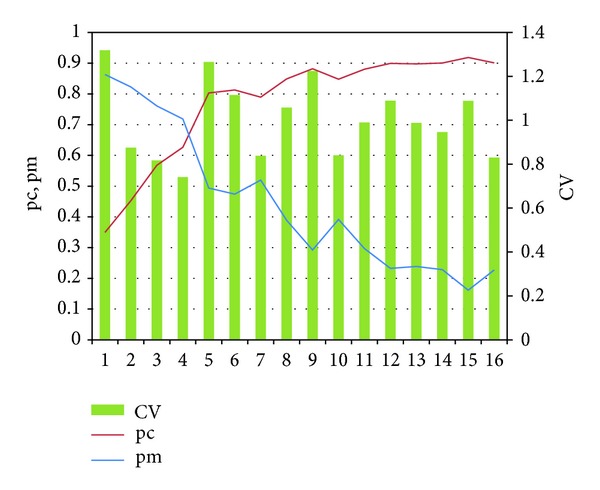
pc, pm, and CV graph over the generation.

Sets of computational experiments have been conducted to explore the behavior of adaptive search strategy. The experiment for 7 nodes sequencing problem is performed and the results are shown in Figure 4.

In the results, we observe that the crossover acceptance probabilities increase according to the number of generations. And when CV of population is high, the acceptance probability of crossover is also high. For example, in Figure 4, the CV of 5th generation is high and so the acceptance probability of crossover is also high. And then the 6th CV is forced to decrease by the increased probability of crossover, which means that the algorithm tries to generate the solutions near the original solutions. When CV is low, the acceptance probability of mutation is high. When the CV at the 7th generation is low, then the acceptance probability of mutation increases and the 8th CV is forced to increase by the increased acceptance probability of mutation. This means that the algorithm generates new solutions far different from the original solutions.

### 5.3. Comparison against Other Algorithms

In this section, the performance of our approach is compared with the optimization technique by OPL-studio (IBM, Available from: http://www.ilog.com/products/optimization/) [23] in terms of the computational time and the quality of solutions. Ten experiments are performed for each problem size on micro-PC with 3.0 GHz processor and 1 GB RAM. The results are shown in Table 3. The initial probabilities are given as pc_0_ = 0.8, 0.7 and pm_0_ = 0.6, 0.7 and *α* = 1 and *β* = 1.

The results show that our algorithm finds the best solution in negligible computation time, while the optimization algorithm requires the considerable computation time and even for 20 nodes problem, it cannot provide a solution in reasonable time.

For the larger size experiments, we use the examples of networks with 25, 35, 45, 70, 85, and 100 nodes. We compared our algorithm with the evolutionary algorithm proposed by Yun and moon [20] in terms of the computing times and the best solution values. The experiments have been performed on micro-PC with 3.0 GHz processor and 2 GB RAM. The results are shown in Table 4.

In Table 4, we observe that two approaches find the best solutions for various examples, but our algorithm is much more efficient in terms of the computing time.

### 5.4. Computational Performance against the Best Known Solutions

TSPLIB problems sets are used to verify the performance of the adaptive evolutionary algorithm. We compare the results of our algorithm to the best known values. We use Sequential Ordering Problem (SOP) set. This problem is an asymmetric traveling salesman problem with additional constraints. Given a set of *n* nodes and distances for each pair of nodes, find a Hamiltonian path from node 1 to node *n* of minimal length which takes given precedence constraints into account. Each precedence constraint requires that node *i* have to be visited before node *j*. The initial probabilities are given as pc_0_ and pm_0_ = 1, *α* = 1, and *β* = 1. And 10∗8 (8 types of option) = 80 times of experiments for each problem were performed to eliminate the random effects.

The results are shown in Table 5. The table shows the % best value to the best known value for the problem set. We used combined options of termination (T), selection (S), and funeral (F) criterion for each problem and compared all the results. On average, 128–131% performances are obtained for all the problems and T1S2F shows the best option. However, we can employ the best option for the specific problem. For example, T1S2 option is the best for the problem *rbg050c*.

In Table 6, the best results obtained in Table 5 are compared with the best known values. We use the selection option 2 in Figure 1 and the first termination criterion with max generation = 10,000. On average, 124% result is obtained. When ESC47 and prob.42 are excluded, the average percentage of best value decreases to 112%.

## 6. Conclusion

In this paper, we presented an adaptive evolutionary algorithm for solving a traveling salesman problem with precedence constraints (TSPPC). Our algorithm employs genetic operators that guarantee the feasibility of solutions, and as the results the searching spaces are significantly reduced. The algorithm also adopts an adaptive search strategy to adaptively apply the crossover and mutation operations on the current generation, which improves the quality of solution as well as the computational efficiency.

The experimental results show that our proposed approach outperforms other evolutionary algorithm in terms of the quality of solution and computation time. Also, we observe that for the small size problems. The algorithm mostly finds the best solution and for even the large size problems, the quality of solution is not much deteriorated and the computation time is quite less. Finally, the proposed algorithm has wide range of options to be applied in various types of problems. We can use combined options of termination, selection of chromosome, and funeral probability.

For future research, our algorithm can be improved in a way to generate more robust solutions, which means that the deviation from a proposed solution by algorithm does not deteriorate the objective function value much so that more practical applications can be possible in real practices.

## Figures and Tables

**Table 1:** Adaptive search strategies.

Factor	Adaptive search strategy	
	Crossover acceptance probability	Mutation acceptance probability
The number of generations	Increase	Decrease
Variance of fitness value		
High	Increase	Decrease
Low	Decrease	Increase

**Table 2:** The performance experiment result 1.

Number of nodes	Parameter		General approach			AEA*		
	*n*	pc_0_	Best solution	Frequency of best	CPU Time (sec)	Best solution	Frequency of best	CPU time (sec)
7	7 14	0.6 0.7	26 26	6 3	0.922 0.015	26 26	1 4	0.006 0.016

25	25 50	0.6 0.7	134 134	10 5	10.422 6.042	134 134	4 5	0.047 0.094

35	30 35 70	0.6 0.7 0.8	177 180 180	11 12 11	18.803 23.844 19.424	177 177 177	9 10 16	0.218 0.235 0.625

45	45 45 50	0.6 0.7 0.8	214 214 209	15 17 18	44.314 38.688 42.124	209 207 207	18 19 17	1.047 1.75 1.547

70	70 70 140	0.6 0.7 0.8	383 372 372	33 21 27	532.406 229.319 245.247	363 363 363	43 40 45	9.016 9.796 14.281

*The proposed adaptive evolutionary algorithm.

**Table 3:** The performance experiment result 2.

Number of nodes	Optimization technique		AEA		
	Optimal solution	CPU Time (sec)	*n*	Best solution	CPU time (sec)
10	18	1.51	25 50	18 18	0.023 0.047
12	27	156.72	25 50	27 27	0.062 0.062
15	49	70626	25 50	49 49	0.088 0.078
20	—	—	25 50	68 73	0.086 0.125

**Table 4:** The performance experiment result 3.

Node	Yun and Moon (2011) [20]						AEA					
	Best value			CPU time			Best value			CPU time		
	Min.	Avg.	Max.	Min.	Avg.	Max.	Min.	Avg.	Max.	Min.	Avg.	Max.
7	26	26.4	28	0.00	0.01	0.01	26	26.4	28	0.00	0.01	0.02
25	134	134.8	141	0.34	0.98	2.56	134	134.2	136	0.31	0.97	2.39
35	177	179.9	186	0.78	5.62	10.84	177	180.3	183	2.36	5.62	11.31
45	209	216.3	224	12.92	29.73	48.66	207	214.9	223	12.64	29.87	57.18
70	368	375.8	386	83.05	146.20	223.80	364	374.5	380	46.38	93.59	161.20
85	420	432.8	471	133.60	326.04	587.30	394	427.3	448	60.22	290.93	467.80
100	458	492.25	520	303.00	676.87	1314.00	448	480.7	505	178.90	491.39	735.60

**Table 5:** Comparison against best known values with TSPLIB problem set.

Name of problem	(%) Best value*/best known								Min
	T1S1**	T1S2	T2S1	T2S2	T1S1F	T1S2F	T2S1F	T2S2F	
br17.10	100%	100%	101%	103%	100%	***100%***	100%	100%	100%
br17.12	100%	100%	100%	103%	100%	***100%***	100%	100%	100%
ESC07	100%	100%	100%	103%	100%	***100%***	100%	100%	100%
ESC12	100%	100%	100%	100%	100%	***100%***	100%	100%	100%
ESC25	142%	155%	144%	150%	**140%**	*156% *	146%	146%	140%
ESC47	363%	373%	365%	351%	378%	*373% *	345%	**323%**	323%
ESC63	119%	120%	125%	122%	122%	***118%***	119%	122%	118%
ft53.1	152%	**141%**	143%	152%	145%	***141%***	144%	145%	141%
ft53.2	141%	138%	132%	138%	137%	***134%***	136%	136%	132%
ft53.3	**121%**	123%	123%	126%	125%	***121%***	122%	125%	121%
ft53.4	122%	**108%**	129%	128%	125%	***108%***	130%	111%	108%
ft70.1	128%	**118%**	121%	120%	125%	*121% *	124%	123%	118%
ft70.2	**115%**	123%	122%	124%	118%	*118% *	118%	125%	115%
p43.1	111%	104%	**103%**	**103%**	107%	***103%***	104%	112%	103%
p43.2	110%	103%	117%	114%	109%	***102%***	111%	108%	102%
p43.3	107%	101%	106%	100%	103%	***102%***	103%	102%	100%
p43.4	106%	101%	107%	106%	102%	***100%***	104%	102%	100%
prob.42	190%	**182%**	197%	196%	192%	*187% *	189%	191%	182%
rbg048a	111%	**107%**	112%	**107%**	108%	***107%***	111%	115%	107%
rbg050c	118%	**108%**	126%	118%	115%	*109% *	120%	117%	108%
ry48p.1	126%	121%	**118%**	123%	122%	*122% *	122%	122%	118%
ry48p.2	121%	**111%**	122%	115%	117%	*116% *	119%	124%	111%
ry48p.3	119%	**113%**	119%	121%	115%	*115% *	118%	124%	113%
ry48p.4	116%	111%	**108%**	**108%**	111%	***108%***	**108%**	112%	108%

avg	**131%**	**128%**	**131%**	**130%**	**130%**	***128%***	**129%**	**129%**	

*The best value obtained by the adaptive evolutionary algorithm.

**T: termination option, 1 = the first option, 2 = the second option.

S: selection option of *n* chromosomes, 1 = the first option, 2 = the second option.

F: funeral option if applied.

**Table 6:** Comparison against best known values using the best options for each problem.

Name of problem	Best	Avg.	Worst	% Best	% Avg.	% Worst	Best known value
br17.10	55	55.9	58	100%	102%	105%	55
br17.12	55	55	55	100%	100%	100%	55
ESC07	2125	2125	2125	100%	100%	100%	2125
ESC12	1675	1720.6	1791	100%	103%	107%	1675
ESC25	2354	3378.4	3972	140%	201%	236%	1681
ESC47	4160	5648.3	6628	**323%**	**439%**	**515%**	1288
ESC63	73	80.9	88	118%	130%	142%	62
ft53.1	10619	11537.4	12323	141%	153%	164%	7531
ft53.2	11000	12092.1	13340	132%	145%	160%	8335
ft53.3	13231	13797	14236	121%	126%	130%	10935
ft53.4	15579	16263.3	16793	108%	113%	116%	14425
ft70.1	46390	48696.2	49623	118%	124%	126%	39313
ft70.2	46485	49162.1	51920	115%	122%	128%	40422
p43.1	28830	29107	29685	103%	104%	106%	27990
p43.2	28896	47979.5	56225	102%	169%	198%	28330
p43.3	28680	29362.5	29555	100%	102%	103%	28680
p43.4	82960	83393.5	83590	100%	101%	101%	82960
prob.42	443	530.7	586	**182%**	**218%**	**241%**	243
rbg048a	376	412.6	435	107%	118%	124%	351
rbg050c	505	528.7	564	108%	113%	121%	467
ry48p.1	18650	21732.2	25057	118%	138%	159%	15805
ry48p.2	18499	22210.8	25969	111%	133%	156%	16666
ry48p.3	22480	25029.2	28744	113%	126%	144%	19894
ry48p.4	33961	34944.7	36421	108%	111%	116%	31446

Average				124%	141%	154%	
Average excluding ESC47 and prob. 42				112%	121%	129%	
